# Validity and Reliability of the Danish Version of the Adult Eating Behavior Questionnaire—Results from the South Danish Obesity Initiative

**DOI:** 10.3390/nu17243824

**Published:** 2025-12-06

**Authors:** Mikkel Emil Iwanoff Kolind, Tobias Midtvedt Windedal, Barbara Vad Andersen, Nina Drøjdahl Ryg, Gabriele Berg-Beckhoff, Claus Bogh Juhl

**Affiliations:** 1 Department of Endocrinology, Esbjerg & Grindsted Hospital, University Hospital of Southern Denmark, 6700 Esbjerg, Denmark; tobias.midtvedt.windedal@rsyd.dk (T.M.W.); claus.bogh.juhl@rsyd.dk (C.B.J.); 2 Department of Regional Health Research, University of Southern Denmark, 5230 Odense, Denmark; 3 Steno Diabetes Center Odense, Odense University Hospital, 5000 Odense, Denmark; 4 OPEN—Open Patient Data Explorative Network, Odense University Hospital, 5000 Odense, Denmark; 5 Department of Food Science, Aarhus University, 8200 Aarhus, Denmark; barbarav.andersen@food.au.dk; 6 Steno Diabetes Center Copenhagen, The Capital Region of Denmark, 2730 Herlev, Denmark; nina.droejdahl.ryg@regionh.dk; 7 Unit for Health Promotion, Department of Public Health, University of Southern Denmark, 6705 Esbjerg, Denmark; gbergbeckhoff@health.sdu.dk

**Keywords:** adult eating behavior questionnaire, eating behavior, appetitive traits, psychometric validation, structural validity, confirmatory factor analysis, test–retest reliability, internal consistency, obesity, Denmark

## Abstract

**Objective**: Appetitive traits influence obesity risk, yet no validated Danish tool exists to assess these traits in adults. We translated the Adult Eating Behavior Questionnaire (AEBQ) into Danish and evaluated reliability and validity. **Methods**: Adults (*n* = 1257) from the South Danish Obesity Initiative completed the Danish AEBQ; a subsample took part in test–retest analysis (*n* = 256). Content validity was assessed via Three-Step Test Interviews (*n* = 5). Test–retest reliability was examined by intraclass correlation (ICC). Confirmatory factor analysis (CFA) tested structural validity (with an ancillary eight- vs. seven-factor comparison). Internal consistency was evaluated by Cronbach’s α and McDonald’s ω. Pearson correlations and regression models (adjusted for age, sex, and education) related subscales to BMI, waist-to-hip ratio (WHR), and body fat percentage (fat%). **Results**: Three-Step Test Interviews supported content validity. Test–retest reliability was good for most subscales (ICCs ≈ 0.80–0.88) and moderate for Emotional Undereating (ICC = 0.640). Both CFA models showed acceptable fit; information criteria favored the seven-factor solution, with small differences on other indices. Internal consistency was acceptable for most subscales (α and ω ≥ 0.70), borderline for Hunger (α = 0.70; ω = 0.71), and below threshold for Satiety Responsiveness (α = 0.69; ω = 0.69). Food Responsiveness and Emotional Overeating were positively associated with BMI/WHR/fat%, while Emotional Undereating showed inverse associations; other subscales showed no associations. **Conclusions**: The Danish AEBQ shows adequate psychometric performance, and both seven- and eight-factor structures appear applicable in a Danish setting, with the caveat that internal consistency for Hunger and Satiety Responsiveness fell just below conventional cut-offs.

## 1. Introduction

Obesity is a major global health challenge, with prevalence rising in nearly every country worldwide [1]. In Denmark, the proportion of adults classed as overweight (BMI > 25 kg/m^2^) increased from 48.8% in 2010 to 52.6% in 2021, while the prevalence of obesity (BMI ≥ 30 kg/m^2^) rose even more sharply, from 13.6% to 18.5% during the same period [2].

Obesity significantly increases the risk of numerous chronic conditions, including type 2 diabetes, obstructive sleep apnea, metabolic dysfunction-associated steatotic liver disease (MASLD), hypertension, and arthritis [3,4,5]. Individuals with class III obesity (BMI ≥ 40 kg/m^2^) have been estimated to have a life expectancy up to ten years shorter than that of normal-weight individuals [6]. In addition, people with obesity tend to experience more sick leave, greater social disadvantage [7], and lower health-related quality of life [8].

The high prevalence and wide-ranging consequences of obesity underscore the need to understand why some individuals are more susceptible to weight gain than others. At its core, obesity results from a persistent imbalance between energy intake and energy expenditure [9]. In high-income countries, where food intake is rarely limited by availability [10], appetite and satiety mechanisms become the primary regulators of food intake. Individual differences in appetitive traits—such as Food Responsiveness (the urge to eat in response to palatable cues) and Satiety Responsiveness (sensitivity to internal fullness signals)—are therefore thought to play a critical role in vulnerability to obesity [11,12]. Despite their relevance, no structured or validated tools currently exist to assess appetite-related traits in Danish adults, limiting the ability to study these mechanisms in national health research or clinical practice.

To study individual variation in appetite traits, several psychometric tools have been developed, including the Child Eating Behavior Questionnaire (CEBQ) and its adult counterpart, the Adult Eating Behavior Questionnaire (AEBQ) [13]. These instruments assess stable, trait-like patterns of appetite-related behavior that influence food intake and preferences across the lifespan. The AEBQ comprises eight subscales, grouped into two broad domains: food approach traits, which are generally associated with increased energy intake and higher BMI, and food avoidance traits, which are typically associated with lower BMI and may serve a protective role by limiting caloric intake [13,14,15,16,17].

However, empirical findings on these associations remain mixed. While several studies report expected links—such as higher food approach scores correlating with greater weight status [13,14,16,17]—others have found no significant associations across most traits [18,19], and some even report inverse findings (e.g., Food Responsiveness negatively associated with BMI) [15,20]. These inconsistencies may reflect cultural, demographic, and/or methodological differences, highlighting the importance of careful translation, adaptation, and validation of the AEBQ for use in specific populations.

The AEBQ has been successfully translated and applied in several countries, including Australia [15], Canada [21], China [18], Norway [20], Mexico [22], Portugal [23], Turkey [24], and Poland [25]. However, questions remain regarding the underlying factor structure of the questionnaire, particularly the role of the Hunger subscale, which captures how often and how strongly individuals experience hunger [13]. Unlike the other scales, which were adapted from the well-established CEBQ, the Hunger scale was newly developed for the AEBQ and has shown inconsistent associations with BMI and other appetitive traits [15,20]. The Hunger subscale has been debated, with some suggesting it overlaps with food responsiveness or reflects a state-like rather than trait-like construct, raising questions about whether it should be retained, merged with other food approach traits, or excluded entirely [15,16,20]. Accordingly, there is ongoing debate about whether a seven- or eight-factor structure provides a better representation of appetitive traits, with several studies reporting improved model fit when the Hunger scale is excluded [15,17,20,21,24] with others favoring the original eight-factor model [13,16,19]. These issues highlight the importance of further investigation into the factor structure and validity of the AEBQ in diverse populations.

The aim of the present study was to translate the AEBQ into Danish and evaluate its psychometric properties in a sample of Danish adults with varying BMI. We assessed content validity, test–retest reliability, internal consistency, and structural validity (factor structure), including a direct comparison of seven- versus eight-factor models (with or without the Hunger subscale). We also examined associations between AEBQ subscales and anthropometric indicators of adiposity (BMI, waist circumference, and body fat percentage).

## 2. Materials and Methods

### 2.1. Participants

Participants were recruited via the South Danish Obesity Initiative (SDOI), a screening program at the University Hospital of Southern Denmark for adults aged 18 to 60 years with BMI ≥ 30 kg/m^2^. The standardized SDOI screening battery, which includes the Adult Eating Behavior Questionnaire, is described in detail elsewhere [26]. To enable comparisons across weight categories, participants without obesity were enrolled within the SDOI framework using the same assessment procedures. Participants with obesity were referred through clinical units, whereas participants without obesity were recruited through nonclinical channels coordinated by SDOI and scheduled for the same visit flow. All participants provided written informed consent for participation and research use of their data (Regional Committee on Health Research Ethics for Southern Denmark: S-20210091). All procedures complied with the Declaration of Helsinki.

### 2.2. Instrument: Adult Eating Behavior Questionnaire

The Adult Eating Behavior Questionnaire is a 35 item self-report instrument with five point Likert responses (1 strongly disagree to 5 strongly agree). Items are grouped into eight subscales: four food approach traits (Hunger, Food Responsiveness, Emotional Overeating, Enjoyment of Food) and four food avoidance traits (Satiety Responsiveness, Emotional Undereating, Food Fussiness, Slowness in Eating). Subscale scores are calculated as the mean of their items after reverse coding where applicable.

### 2.3. Translation Procedure

The AEBQ was translated from English to Danish using a forward–backward procedure. Three forward translators (one dietitian and two health researchers without formal training in dietetics), all native Danish speakers with university-level English proficiency, produced a forward translation and reconciled it by consensus. Two back-translators (a nurse and a school teacher), blinded to the original and without prior experience with dietary screening tools, then back-translated the reconciled Danish version into English. The school teacher was a native English speaker fluent in Danish, and the nurse was a native Danish speaker fluent in English.

The full translation team compared the source instrument, the reconciled Danish translation, and the back-translation; documented discrepancies; and resolved them to produce a pre-final Danish version. The pre-final version was pilot-tested in 11 respondents to ensure feasibility. Participants were asked to note any errors or difficulties: none were reported. Based on the pilot-test no further changes were needed, and the translated Danish version of the AEBQ was adopted unchanged. Original and Danish wording for all items and scale values are presented in Appendix A (Table A1 and Table A2).

Readability of the finalized Danish questionnaire, assessed using the LIX readability index [27], corresponds to approximately a Danish 6th-grade reading level (~12 years).

### 2.4. Content Validity Assessment: Three-Step Test Interview

To evaluate content validity and comprehensibility of the translated questionnaire, we used the Three-Step Test Interview (TSTI) method [28]. One-on-one interviews were conducted in a quiet room at the SDOI clinic. In Step 1, participants completed the questionnaire while thinking aloud, verbalizing their thoughts as they read and answered each item. In Step 2, the interviewer used focused probes to elicit elaboration on observed utterances or behaviors during completion, without encouraging retrospective reasoning. In Step 3, participants reflected on the questionnaire as a whole and suggested improvements. All interviews were audio-recorded and transcribed for analysis. Five participants were invited for the TSTI as a targeted assessment of relevance, comprehensibility, and comprehensiveness [29].

### 2.5. Test–Retest Reliability

To assess test–retest reliability of the Danish AEBQ, we aimed to recruit ≥200 participants from the SDOI cohort who had previously completed the full SDOI screening battery. With *n* = 200 and two administrations, an ICC of 0.75 (two-way random-effects, absolute agreement, single measures) was expected to yield a 95% CI half-width of ~±0.06–0.08 (α = 0.05), which we judged to be adequate precision. Eligible participants were informed about the purpose and procedures and, upon consent, received a secure REDCap link to the questionnaire; 14 days later, a second link was sent for re-administration. Non-responders (completed the first but not the second) received an automatic email reminder on day 21, a phone reminder on day 22 if still pending, and a final email reminder on day 30. For all participants, we recorded the inter-administration interval and completion time at each administration.

### 2.6. Baseline Characteristics

Sociodemographic information, anthropometric, and body composition measurements collected as part of the SDOI screening program were included. Sociodemographic information included education level, employment status, and marital status. Anthropometrics comprised measured weight, height, waist circumference, and hip circumference obtained by trained clinical staff. Body composition was measured using multi-frequency bioelectrical impedance analysis (InBody 770; InBody Co., Seoul, Republic of Korea). BMI was calculated as weight (kg)/height (m)^2^ and waist-to-hip ratio as waist/hip (cm).

### 2.7. Statistical Analyses

Test–retest reliability was assessed using intraclass correlation coefficients (ICC), based on a two-way random-effects model for absolute agreement and single measures [30]. We interpreted ICC values according to Koo and Li (2016): values < 0.5 indicate poor reliability, 0.5–0.75 moderate, 0.75–0.90 good, and >0.90 excellent reliability [31].

SEM and minimal detectable change (MDC) were calculated as indicators of measurement error. Both are reported in scale units (Likert points), with smaller values reflecting greater measurement precision.

Confirmatory factor analysis (CFA) was used to evaluate the internal structure of the AEBQ. Two a priori models were tested based on prior work [13,15,22]: (i) the original 8-factor, 35-item structure and (ii) a 7-factor, 30-item structure excluding the Hunger subscale. Model fit was summarized with comparative fit index (CFI), Tucker–Lewis index (TLI), root mean squared error of approximation (RMSEA), standardized root mean squared residual (SRMR), and χ^2^/df. Acceptable fit was defined as CFI/TLI ≥ 0.90, RMSEA ≤ 0.06, and SRMR ≤ 0.08 [30]. For model comparison, the Akaike information criterion (AIC) and Bayesian information criterion (BIC) were also considered (lower values indicating better fit).

Internal consistency was assessed for each subscale using Cronbach’s α. McDonald’s omega coefficients (ω) were computed as a secondary measure of internal consistency to account for differential item loadings. We interpreted reliability (α, ω) ≥ 0.70 as acceptable [32]. For preliminary support of the factorability of the data and to justify model specification, we also report standardized factor loadings for each item.

Associations between AEBQ subscale scores and body composition measures (waist and hip circumference, BMI, fat percentage, and waist-to-hip ratio) were examined using Pearson’s correlations, stratified by sex. Correlations between different AEBQ subscales were similarly assessed. In addition, multivariable linear regression analyses were run one subscale at a time for each body composition outcome, with adjustment for age, sex, and education. *p*-values < 0.05 were considered statistically significant.

## 3. Results

### 3.1. Participant Charectaristics

After excluding participants who did not consent to research use of their data (*n* = 37), data were available for 1257 participants in the SDOI cohort. Data were collected from September 2020 to June 2025. The test–retest survey was administered from July 2023 to February 2024. The SDOI cohort and the test–retest sample were similar on all characteristics except age; the test–retest sample was, on average, 2.9 years older (Table 1).

### 3.2. Content Validity

Results from the TSTIs indicated that participants understood the Danish translation of the AEBQ as intended and found the items personally meaningful. Responses showed that the item wording elicited reflective and construct-relevant elaborations, supporting comprehension, and relevance. Some variation in interpretation was observed for items such as “I love food” and “I eat more when I’m anxious,” and two participants noted potential missing items related to tiredness and boredom as triggers for eating. These nuances suggest minor limitations in comprehensiveness, but overall the instrument appeared conceptually aligned and functioned as intended (Supplementary File S1).

### 3.3. Test–Retest Reliability

The AEBQ took a median of 210 s (IQR 168–270) at the first administration and 213 s (IQR 165–274) at the second administration. Completion times ranged from 75 to 12,468 s at the first administration and from 55 to 12,740 s at the second, with the extreme upper values likely reflecting participants who paused and did not complete the questionnaire in one sitting. The median interval between completions was 16 days (IQR 14–21).

ICC values were consistently in the good range for the two higher-order dimensions (food approach and food avoidance) regardless of whether the Hunger subscale was included or not. Emotional Undereating showed only moderate reliability, while all other subscales reached good reliability according to established benchmarks.

With the exception of Emotional Overeating and Emotional Undereating all MDC values were below 1 (Table 2), indicating that a one-point change exceeds measurement error for non-emotional eating subscales. In a supplementary sensitivity analysis, data was restricted to participants with ≤16 days between completion. Results were similar although the ICC for Emotional Undereating changed to >0.7 (from 0.640 to 0.716) (see Appendix B, Table A3).

### 3.4. Confirmatory Factor Analysis

Both the eight-factor model including all 35 items (Model 1) and the seven-factor model excluding the Hunger scale (Model 2; 30 items) demonstrated acceptable overall fit according to conventional criteria. Comparative fit indices were marginally better in Model 2 (CFI = 0.923, TLI = 0.913) than in Model 1 (CFI = 0.917, TLI = 0.907), indicating a slight improvement in relative fit when the Hunger scale was omitted. RMSEA values were acceptable and similar across models (0.052 for Model 1 and 0.056 for Model 2), suggesting adequate approximation to population covariance structures in both solutions, with a slightly better fit for Model 1.

SRMR was marginally lower for Model 2 than Model 1 (0.051 vs. 0.053), indicating that this model reproduced observed pairwise correlations slightly more closely on average. However, this difference was minimal and well below the conventional threshold for good fit (≤0.08), suggesting that both models performed equivalently on this criterion.

Information criteria metrics favored model 2, with lower AIC (93,129 vs. 110,476) and BIC (93,698 vs. 111,158), reflecting model 2’s more parsimonious structure (Table 3).

### 3.5. Internal Consistency and Factor Loadings

Cronbach’s α and McDonald’s ω were close to but slightly below the acceptable range for the Hunger subscale, and both indices fell below the 0.70 threshold for the Satiety Responsiveness subscale. Within the Hunger subscale, several items showed relatively low factor loadings (<0.50), which could contribute to its reduced reliability (e.g., Q6 and Q34—see Appendix A, Table A2 for item descriptions). For the remaining subscales, internal consistency indices were acceptable to excellent, and standardized factor loadings in both the eight- and seven-factor models were generally moderate to high, supporting the intended structure of the questionnaire (Table 4).

### 3.6. Associations Between Subscales and Adiposity Measures

AEBQ subscales were generally intercorrelated in expected directions, with the strongest associations observed among the food approach scales. The food avoidance subscales were also moderately interrelated. Regarding anthropometric measures, small but significant correlations emerged: Food Responsiveness and Emotional Overeating correlated positively with BMI and body fat percentage, while Emotional Undereating correlated negatively, in line with theoretical expectations. In contrast, several associations deviated from expectations. Food Fussiness was positively associated with both BMI and body fat percentage, while Satiety Responsiveness and Slowness in Eating were also positively associated with adiposity measures, opposite to the anticipated direction (Table 5).

**Figure 1:**
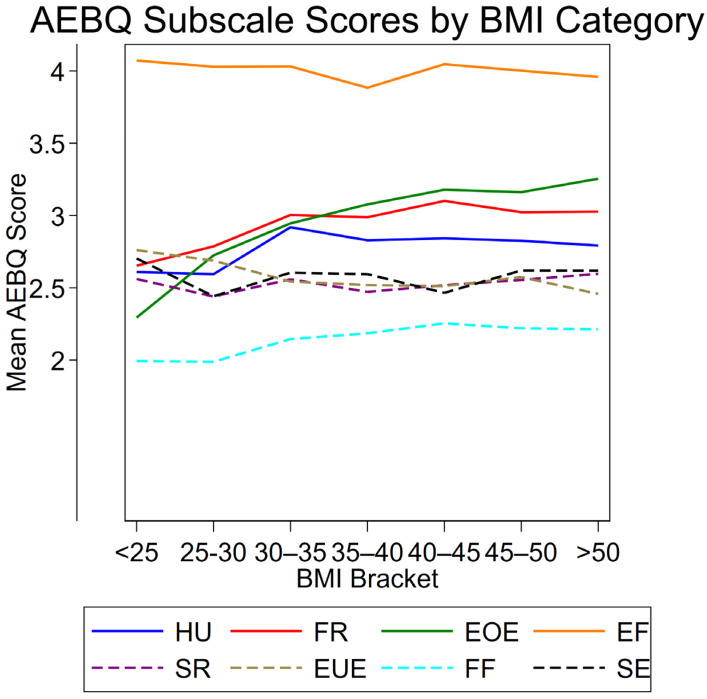
Mean AEBQ subscale scores across BMI brackets. Solid lines represent food approach subscales, while dashed lines represent food avoidance subscales. HU = Hunger; FR = Food Responsiveness; EOE = Emotional Overeating; EF = Enjoyment of Food; SR = Satiety Responsiveness; EUE = Emotional Undereating; FF = Food Fussiness; SE = Slowness in Eating. BMI = Body Mass Index.

To help visualize the relationship between eating behavior traits and adiposity measures, the mean AEBQ subscale scores were plotted across BMI brackets in five-point increments (Figure 1). The largest differences in subscale scores appeared in the lower BMI categories (<25 to 30–35), with particularly pronounced increases for Hunger, Food Responsiveness, and Emotional Overeating. Enjoyment of Food and the food avoidance scales showed only minor variation across BMI brackets. Notably, Emotional Overeating differed from the other approach scales by displaying a steady decline across the lowest three BMI brackets. Overall, Figure 1 illustrates that the strongest variation in eating behavior traits was concentrated in the lower BMI ranges, while differences leveled off at higher BMI.

After adjusting for age, sex, and education, Food Responsiveness and Emotional Overeating were positively associated with BMI, waist-to-hip ratio, and body fat percentage. Emotional Undereating showed inverse associations with BMI and body fat percentage, while Food Fussiness was positively associated with body fat percentage only. No adjusted associations were observed for Hunger, Enjoyment of Food, Satiety Responsiveness, or Slowness in Eating (Table 6).

## 4. Discussion

This study evaluated the Danish translation of the AEBQ and provides evidence supporting its content validity, test–retest reliability, structural validity, and internal consistency, in a Danish cohort with a broad BMI range. Overall, the questionnaire functioned as intended, with most subscales demonstrating acceptable psychometric properties and some intercorrelations in line with theoretical expectations. As have been noted in previous validations [15,17,20,21,24], our findings also highlight some challenges, particularly with the Hunger and Satiety Responsiveness subscales.

Cognitive interviews indicated that the Danish AEBQ was generally well understood and perceived as relevant, with only minor nuances in interpretation (Supplementary File S1). Consistent with this, completion time was brief—median 210–213 s across administrations—suggesting low respondent burden and good feasibility in routine data collection.

In terms of reliability, our results showed good test–retest stability for most AEBQ subscales, with ICCs indicating good reliability. The exception being the Emotional Undereating subscale with an ICC < 0.75 indicating a moderate level of stability. The reliability of the AEBQ subscales were similarly confirmed in other studies [13,16,18,33]. We also report measurement error indices: MDC values with <1 Likert point for all traits except the two emotional subscales (Emotional Undereating MDC = 1.419; Emotional Overeating MDC = 1.195). Thus, for all non-emotional traits, a change of ≥1 point likely reflects real change, whereas Emotional Overeating and Emotional Undereating appear to require a change greater than 1 point to exceed measurement error. This suggests that emotional aspects of eating are more state-like and prone to short-term fluctuation, while the remaining AEBQ traits behave more stably over time.

We wanted to examine the structural validity of the Danish version of the AEBQ, with particular attention to the role of the Hunger subscale. Consistent with earlier validation studies, our analyses showed that both the original eight-factor structure and alternative models excluding the Hunger subscale demonstrated an acceptable fit [15,22,33]. Removing the Hunger subscale yielded small improvements on most fit indices, echoing previous reports [14,15,16,17,19,20,21,23,33]. Notably, the eight-factor model exhibited a slightly lower RMSEA than the seven-factor model—a finding observed by others [16,21]—which is plausible given that RMSEA tends to penalize model complexity less strongly than comparative indices such as CFI and TLI. Taken together, our findings indicate that for the Danish AEBQ, both the eight- and seven-factor solutions perform adequately, with the seven-factor model offering greater parsimony and the eight-factor model showing marginally better approximation fit.

When viewed in an international context, the Danish AEBQ performs similarly to other translated versions with respect to the seven- and eight-factor structures. In Australian, Canadian, and UK samples [15,21,33], both the original eight-factor model and the seven-factor solution excluding Hunger generally reached CFI ≥ 0.90 and RMSEA ≤ 0.06, with the seven-factor model typically showing slightly higher CFI/TLI than the eight-factor model. In Chinese and French-Canadian adult samples [16,18], both seven- and eight-factor solutions showed very strong fit (CFI/TLI > 0.97 and RMSEA ≈ 0.03), whereas some adolescent validations from Norway and Portugal [20,23] reported CFIs below 0.90 and RMSEA values around 0.07, which would fall outside our predefined cut-offs. Against this backdrop, our Danish eight- and seven-factor models (CFI ≈ 0.92, TLI ≈ 0.91, RMSEA ≈ 0.05–0.06) sit in the mid-range of international findings: stronger than several non-English-speaking adolescent validations (e.g., Norway and Portugal), broadly comparable to other adult community samples, but not as close-fitting as the best-performing East Asian and French-Canadian models. Across these studies, Hunger (and, in some cases, Satiety Responsiveness) shows the weakest internal consistency, often just below 0.70 (e.g., [15,16,20,21,33]), reinforcing that the challenges we observe for these subscales in Denmark reflect a recurrent feature of the adult AEBQ rather than a uniquely Danish phenomenon.

In addition to this recurrent reliability pattern, Hunger also shows a characteristic association profile across studies. In our data and in several other adult validations, the Hunger subscale correlated moderately with other food approach traits [13,15,16,18,21,33,34], yet showed no clear associations with BMI [15,16,21]. Jacob et al. [16] propose that, in adults, the current Hunger items may predominantly capture very intense, late-stage hunger sensations (e.g., feeling lightheaded or irritable, needing to eat “right away”), rather than a nuanced awareness of early or moderate hunger signals. In Jacob et al. [16], the AEBQ Hunger subscale was positively correlated with the Three-Factor Eating Questionnaire’s Susceptibility to Hunger subscale, particularly items reflecting an internal locus of hunger signals, indicating that it taps genuine experiences of hunger, while at the same time, similar to the present study, showing slightly sub-threshold internal consistency, weak or absent associations with BMI, as well as negative associations with intuitive eating and “eating for physical rather than emotional reasons”. Taken together, Jacob et al. argue that the Hunger scale may rather represent experiencing very strong hunger sensations, which could reflect a lack of awareness or responsiveness to more subtle or adequate hunger sensations, and that it may characterize a maladaptive form of eating regulation rather than a direct risk factor for obesity. Our Danish findings are compatible with this view and suggest that, in adult samples at elevated cardiometabolic risk, Hunger may function as a relatively state-sensitive, yet reasonably stable, pattern of intense hunger experiences, as indicated by the good test–retest reliability reported in our study and previous validations [13,16,18,22,33].

Overall, we do not consider the current evidence sufficient to reject the original eight-factor structure. Instead, we recommend that researchers retain the full item set but interpret the Hunger subscale with particular caution, given its comparatively lower internal consistency and likely greater sensitivity to contextual influences. In line with Jacob et al., future studies should further examine the relationship between Hunger scores, objectively measured energy intake, and eating-disorder symptoms (e.g., binge eating), to clarify whether and when the subscale indexes show a clinically relevant vulnerability to overconsumption rather than benign variability in hunger experiences [16].

Our analyses of inter-subscale correlations were broadly consistent with theoretical expectations [13], with the strongest associations observed among the food approach traits and more moderate clustering of the avoidance traits. The only exception was a positive association between Food Fussiness, an avoidance trait, and Emotional Overeating, an approach trait. Similar findings have been reported by others [13,15], while most studies report no association between Food Fussiness and Emotional Overeating [16,17,25,33]. Originally developed in the CEBQ, Food Fussiness conceptually indexes selective, sensory-driven food choice rather than caloric drive [11]. This may protect against adiposity when it limits intake/variety in childhood [35], but expose to risk in adulthood when the same trait channels intake toward palatable, less healthy options or emotionally driven eating [13,36].

When eating behavior traits were linked to anthropometric outcomes, findings were mixed. As anticipated, Food Responsiveness and Emotional Overeating showed positive associations with BMI, WHR, and fat%, while Emotional Undereating was inversely associated with BMI and fat%; other associations were non-significant. Similar findings were reported by Zickgraf et al. (2018) [19], who tested the factor structure of the AEBQ in bariatric surgery candidates: like us, they found a positive Emotional Overeating–BMI association and a negative Emotional Undereating–BMI association, but no association between Food Responsiveness and BMI. This is plausibly explained by restricted BMI variability (ceiling effects) in Zickgraf’s exclusively high-BMI cohort. Although our cohort was not exclusively obese, the majority had obesity, which likely attenuated associations in our data as well (Figure 1 indicates ceiling effects) while the inclusion of composition-sensitive outcomes (fat% and WHR) provided additional detectable signals. Overall, the evidence supports Food Responsiveness/Emotional Overeating as risk-oriented traits and Emotional Under Eating as protective even in high-adiposity contexts, whereas the small positive associations of Food Fussiness, Satiety Responsiveness, and Slowness in Eating with adiposity run counter to the original theoretical model. Several contextual explanations are plausible. In this predominantly high-adiposity sample, ceiling effects in BMI may distort linear associations for traits usually regarded as “protective” in leaner populations, and the preventive cardiometabolic setting likely attracted participants who are already weight-concerned and actively trying to change how they eat (e.g., slowing down, paying attention to fullness). Moreover, completing an eating behavior questionnaire in this context may promote socially desirable responding (endorsing items that sound healthy, such as being selective or eating slowly), which may not fully reflect habitual intake [37]. Notably, our study is among the first AEBQ validations to include body fat percentage, and the positive association between Food Fussiness and fat%, but not BMI, may indicate that, in adults, a more selective eating style steers choices towards a narrow range of energy-dense “safe” foods [13,36] that may impact body composition more than weight alone. However, the unexpected positive associations for these avoidance traits may reflect features of this specific sample and measurement context, and further research is needed before extrapolating to other adult populations.

### Strengths and Limitations

A key strength of this study is the relatively large sample size, which provided sufficient power for CFA and reliability analyses and allowed us to examine associations with multiple anthropometric and body composition outcomes. In addition, we used a multi-method psychometric approach, combining content validity evaluation (TSTI), internal consistency indices (α and ω), test–retest reliability with SEM/MDC, and confirmatory factor analysis. Several limitations should also be acknowledged. First, we did not perform formal measurement invariance analyses across sex or BMI categories. Our CFA results therefore pertain to the pooled sample, and we cannot empirically confirm that the factor structure is identical across subgroups. Second, as this is a population with a high degree of somatic and psychiatric comorbidities [26,38], we cannot exclude residual confounding. Although our regression models were adjusted for age, sex, and education, we did not account for factors such as overall disease burden, use of psychotropic or other weight-affecting medications, or ongoing lifestyle interventions; accordingly, the observed associations between appetitive traits and anthropometric outcomes should be interpreted with caution. Third, our content validity evaluation (TSTI) was based on only five participants, all recruited from the same clinical program. While this sample size is acceptable within COSMIN guidance [29], it necessarily limits the breadth of perspectives captured and the strength of the evidence. Finally, the cross-sectional design and reliance on self-reported eating behavior constrain causal inference and may be affected by social desirability and recall bias, particularly in a preventive cardiometabolic setting.

## 5. Conclusions

The Danish AEBQ showed adequate psychometric performance: good content validity, generally good test–retest reliability (with Emotional Undereating less stable), acceptable structural validity for both eight- and seven-factor models (the latter more parsimonious), and acceptable internal consistency (borderline for Hunger; below threshold for Satiety Responsiveness). In adjusted analyses, Food Responsiveness and Emotional Overeating tracked positively with adiposity, whereas Emotional Undereating tracked negatively; other subscales showed small or no associations. Taken together, our findings support the use of the Danish AEBQ, while suggesting some caution when interpreting the emotional subscales and the Hunger and Satiety Responsiveness scales. Future work in Danish samples should clarify the clinical utility of the AEBQ, including whether appetitive profiles add value beyond standard anthropometric and clinical indicators.

## Figures and Tables

**Table 1:** Participant characteristics.

Characteristic	SDOI Cohort (*n* = 1257)	Test–Retest Group (*n* = 256)
Sex (female), *n* (%)	895 (71.2)	184 (71.9)
Ethnicity (Danish), *n* (%)	1166 (92.8)	244 (95.3)
Living arrangement:		
Living with others, *n* (%)	955 (76.0)	197 (77.0)
Living alone, *n* (%)	301 (24.0)	59 (23.0)
Highest achieved education:		
Primary school, *n* (%)	209 (16.6)	40 (15.6)
Secondary education, *n* (%)	210 (16.7)	31 (12.1)
Medium-length, *n* (%)	661 (52.6)	133 (52.0)
University, *n* (%)	109 (8.7)	22 (8.6)
No answer provided, *n* (%)	68 (5.4)	30 (11.7)
BMI categories		
<25 kg/m^2^	111 (8.8)	26 (10.2)
25–30 kg/m^2^	103 (8.2)	30 (11.7)
30–35 kg/m^2^	182 (14.5)	34 (13.3)
35–40 kg/m^2^	311 (24.7)	57 (22.3)
40–45 kg/m^2^	265 (21.1)	47 (18.4)
45–50 kg/m^2^	163 (13.0)	39 (15.2)
>50 kg/m^2^	122 (9.7)	23 (9.0)
Age, mean (SD), years	42.2 (11.1)	45.1 (10.8) *
Height, mean (SD), cm	171.5 (9.2)	170.9 (8.9)
Weight, mean (SD), kg	113.8 (28.7)	111.2 (28.0)
BMI, mean (SD), kg/m^2^	38.7 (9.0)	38.1 (9.3)
Waist circumference, mean (SD), cm	117.6 (20.7)	115.8 (21.4)
Body fat percentage, mean (SD), %	126.5 (17.7)	125.8 (18.5)

Values are n (%) or mean (SD). Test–retest group is a subsample of the SDOI cohort. SDOI = South Danish Obesity Initiative; BMI = Body Mass Index. * *p* < 0.001 vs. SDOI cohort (Welch *t*-test).

**Table 2:** Test–retest reliability.

	Test 1	Test 2			
	Mean (SD)	Mean (SD)	SEM	MDC	ICC (95% CI)
Food Approach (excl. Hunger)	3.04 (0.64)	3.03 (0.67)	0.26	0.71	0.877 (0.846; 0.903)
Food Approach (incl. Hunger)	3.17 (0.71)	3.16 (0.74)	0.22	0.62	0.885 (0.856; 0.910)
Food Approach Subscales					
HU	2.73 (0.72)	2.73 (0.72)	0.324	0.898	0.798 (0.750; 0.839)
FR	2.82 (0.82)	2.86 (0.78)	0.327	0.907	0.832 (0.791; 0.867)
EOE	3.01 (1.00)	2.96 (1.03)	0.431	1.195	0.820 (0.776; 0.857)
EF	3.90 (0.80)	3.89 (0.79)	0.326	0.905	0.831 (0.790; 0.866)
Food Avoidance	2.50 (0.54)	2.53 (0.53)	0.23	0.64	0.815 (0.770; 0.853)
Food Avoidance Subscales					
SR	2.79 (0.86)	2.81 (0.81)	0.350	0.971	0.823 (0.780; 0.859)
EUE	2.49 (0.84)	2.57 (0.86)	0.512	1.419	0.640 (0.565; 0.709)
FF	2.22 (0.90)	2.21 (0.84)	0.304	0.842	0.878 (0.847; 0.904)
SE	2.59 (0.97)	2.62 (0.94)	0.360	0.997	0.857 (0.822; 0.887)

Values are mean (SD) at Test 1 and Test 2. SEM = standard error of measurement; MDC = minimal detectable change; ICC = intraclass correlation coefficient; CI = confidence interval. HU = Hunger; FR = Food Responsiveness; EOE = Emotional Overeating; EF = Enjoyment of Food; SR = Satiety Responsiveness; EUE = Emotional Undereating; FF = Food Fussiness; SE = Slowness in Eating.

**Table 3:** Confirmatory factor analysis.

Model	Items	Factors	CFI	TLI	RMSEA	SRMR	χ^2^ (df)	AIC	BIC
1	35	8	0.917	0.907	0.052 (0.050; 0.054)	0.053	2318.3 (532)	110,476	111,158
2	30	7	0.923	0.913	0.056 (0.053; 0.058)	0.051	1875.2 (384)	93,129	93,698

Model 1 = original eight-factor, 35-item structure; Model 2 = seven-factor, 30-item structure excluding the Hunger scale. CFI = comparative fit index; RMSEA = root mean square error of approximation with 90% confidence interval in parentheses; SRMR = standardized root mean square residual; χ^2^ (df) = chi-square statistic with degrees of freedom in parentheses; AIC/BIC = Akaike/Bayesian information criteria.

**Table 4:** Internal consistency and factor loading.

				Eight-Factor Model	Seven-Factor Model
Factor	Item	α	ω	λ (95% CI)	λ (95% CI)
HU	Q6	0.6995	0.7065	0.375 (0.321; 0.429)	-
	Q9			0.492 (0.444; 0.541)	-
	Q28			0.728 (0.693; 0.762)	-
	Q32			0.789 (0.758; 0.820)	-
	Q34			0.391 (0.338; 0.445)	-
FR	Q13	0.7823	0.7841	0.633 (0.595; 0.670)	0.618 (0.578; 0.658)
	Q17			0.775 (0.746; 0.804)	0.795 (0.766; 0.823)
	Q22			0.762 (0.733; 0.791)	0.761 (0.731; 0.792)
	Q33			0.604 (0.564; 0.644)	0.596 (0.555; 0.638)
EOE	Q5	0.9062	0.9066	0.815 (0.794; 0.837)	0.816 (0.794; 0.837)
	Q8			0.877 (0.861; 0.893)	0.878 (0.862; 0.894)
	Q10			0.906 (0.892; 0.920)	0.906 (0.892; 0.919)
	Q16			0.753 (0.727; 0.780)	0.753 (0.727; 0.780)
	Q21			0.706 (0.675; 0.736)	0.705 (0.675; 0.736)
EF	Q1	0.8287	0.8357	0.786 (0.758; 0.815)	0.785 (0.757; 0.814)
	Q3			0.823 (0.797; 0.850)	0.823 (0.797; 0.849)
	Q4			0.773 (0.743; 0.803)	0.774 (0.744; 0.803)
SR	Q11	0.6874	0.6922	0.488 (0.435; 0.541)	0.495 (0.443; 0.548)
	Q23			0.519 (0.468; 0.569)	0.522 (0.471; 0.572)
	Q30			0.616 (0.571; 0.662)	0.621 (0.575; 0.666)
	Q31			0.755 (0.714; 0.796)	0.746 (0.705; 0.787)
EUE	Q15	0.8935	0.8962	0.783 (0.758; 0.808)	0.783 (0.757; 0.808)
	Q18			0.716 (0.686; 0.747)	0.715 (0.685; 0.746)
	Q20			0.866 (0.848; 0.885)	0.866 (0.848; 0.885)
	Q27			0.854 (0.834; 0.873)	0.854 (0.835; 0.874)
	Q35			0.763 (0.736; 0.790)	0.763 (0.736; 0.790)
FF	Q2	0.8701	0.8717	0.620 (0.582; 0.659)	0.620 (0.582; 0.659)
	Q7			0.692 (0.658; 0.725)	0.692 (0.659; 0.725)
	Q12R *			0.873 (0.854; 0.892)	0.873 (0.854; 0.892)
	Q19R *			0.841 (0.820; 0.863)	0.841 (0.820; 0.863)
	Q24R *			0.752 (0.724; 0.780)	0.752 (0.724; 0.780)
SE	Q14R *	0.8362	0.8441	0.757 (0.729; 0.786)	0.753 (0.723; 0.782)
	Q25			0.787 (0.760; 0.815)	0.788 (0.760; 0.815)
	Q26			0.553 (0.511; 0.595)	0.554 (0.511; 0.596)
	Q29			0.913 (0.893; 0.934)	0.913 (0.893; 0.934)

α = Cronbach’s alpha; ω = McDonald’s omega; λ = standardized item loading. HU = Hunger; FR = Food Responsiveness; EOE = Emotional Overeating; EF = Enjoyment of Food; SR = Satiety Responsiveness; EUE = Emotional Undereating; FF = Food Fussiness; SE = Slowness in Eating. * *p* < 0.05.

**Table 5:** Bivariate associations between AEBQ subscales and adiposity measures.

	FR	EOE	EF	SR	EUE	FF	SE	BMI	WHR	Fat%
HU	0.60 ***	0.42 ***	0.31 ***	−0.18 ***	−0.02	0.02	−0.08 **	0.04	−0.03	0.10 ***
FR		0.56 ***	0.53 ***	−0.33 ***	−0.16 ***	−0.03	−0.16 ***	0.11 ***	0.03	0.12 ***
EOE			0.23 ***	−0.19 ***	−0.31 ***	0.07 *	−0.07 **	0.22 ***	−0.03	0.27 ***
EF				−0.34 ***	−0.11 ***	−0.32 ***	−0.13 ***	−0.03	0.05	−0.09 **
SR					0.32 ***	0.18 ***	0.29 ***	0.03	−0.08 **	0.12 ***
EUE						0.04	0.15 ***	−0.08 **	−0.04	−0.06 *
FF							0.03	0.08 **	−0.01	0.14 ***
SE								−0.01	−0.12 ***	0.08 **

Bivariate associations between AEBQ subscales and adiposity measures estimated with Pearson correlation coefficients. HU = Hunger; FR = Food Responsiveness; EOE = Emotional Overeating; EF = Enjoyment of Food; SR = Satiety Responsiveness; EUE = Emotional Undereating; FF = Food Fussiness; SE = Slowness in Eating. BMI = Body Mass Index; WHR = waist-to-hip ratio; Fat% = body fat percentage. * *p* < 0.05, ** *p* < 0.01, *** *p* < 0.001.

**Table 6:** Multiple linear regression results for associations between AEBQ subscales and adiposity measures.

Dimension	BMI β (*p*)	WHR β (*p*)	Fat% β (*p*)
Food approach scales			
HU	0.251	0.004	0.606
FR	1.100 ***	0.013 ***	1.086 ***
EOE	2.014 ***	0.009 ***	2.119 ***
EF	−0.236	0.004	−0.666
Food avoidance scales			
SR	−0.005	0.000	0.425
EUE	−1.143 ***	−0.002	−1.118 ***
FF	0.422	0.003	1.003 **
SE	−0.212	−0.004	−0.044

Linear regression analyses examining associations between AEBQ subscales and anthropometric measures (BMI, waist-hip ratio, and body fat percentage) as dependent outcomes, adjusted for age, sex, and education. Values are unstandardized regression coefficients (β). HU = Hunger; FR = Food Responsiveness; EOE = Emotional Overeating; EF = Enjoyment of Food; SR = Satiety Responsiveness; EUE = Emotional Undereating; FF = Food Fussiness; SE = Slowness in Eating. BMI = Body Mass Index; WHR = waist-to-hip ratio; Fat% = body fat percentage. ** *p* < 0.01, *** *p* < 0.001

## Data Availability

Data may be made available upon reasonable request by contacting the corresponding author.

## Supplementary Materials

The following supporting information is available online at the journal website: https://www.mdpi.com/article/10.3390/nu17243824/s1. Supplementary File S1: Three-Step Test Interview: Overview and Focus Areas.

## Abbreviations

The following abbreviations are used in this manuscript:

**Abbrev.**	**Meaning**
AEBQ	Adult Eating Behavior Questionnaire
AIC	Akaike information criterion
BIC	Bayesian information criterion
BMI	Body Mass Index
CEBQ	Child Eating Behavior Questionnaire
CFA	Confirmatory factor analysis
CFI	Comparative fit index
CI	Confidence interval
EOE	Emotional Overeating (AEBQ subscale)
EF	Enjoyment of Food (AEBQ subscale)
EUE	Emotional Undereating (AEBQ subscale)
FF	Food Fussiness (AEBQ subscale)
FR	Food Responsiveness (AEBQ subscale)
HU	Hunger (AEBQ subscale)
ICC	Intraclass correlation coefficient
LIX	Læse IndeX (readability index)
MASLD	Metabolic dysfunction–associated steatotic liver disease
MDC	Minimal detectable change
OPEN	Open Patient data Explorative Network
RMSEA	Root mean square error of approximation (90% CI)
SD	Standard deviation
SE	Slowness in Eating (AEBQ subscale)
SDOI	South Danish Obesity Initiative
SR	Satiety Responsiveness (AEBQ subscale)
SRMR	Standardized root mean square residual
TLI	Tucker–Lewis index
TSTI	Three-Step Test Interview
WHR	Waist-to-hip ratio

## Appendix A

Table A1 presents the original English wording of the Adult Eating Behavior Questionnaire (AEBQ) items alongside their Danish translations.

**Table A1:** Original and translated title, instruction, and scale values.

Danish title	Spørgeskema om spiseadfærd hos voksne				
Original English title	Adult Eating Behavior Questionnaire				
Danish instruction	Læs venligst hvert udsagn og sæt kryds ved det udsagn der passer bedst for dig				
Original English instruction	Please read each statement and tick the response that best reflects how much you agree or disagree				
Response option and scale values:					
Code	1	2	3	4	5
Danish label	Meget uenig	Uenig	Hverken enig eller uenig	Enig	Meget enig
English label	Strongly disagree	Disagree	Neither agree nor disagree	Agree	Strongly agree

**Table A2:** Original and translated wording in the AEBQ. All items.

Item	Subscale	Danish Wording	Original English Wording
1	Enjoyment of Food	Jeg elsker mad	I love food
2	Food Fussiness	Jeg beslutter ofte at jeg ikke kan lide maden inden jeg smager på den	I often decide that I don’t like a food before tasting it
3	Enjoyment of Food	Jeg nyder at spise	I enjoy eating
4	Enjoyment of Food	Jeg ser frem til spisetiderne	I look forward to mealtimes
5	Emotional Overeating	Jeg spiser mere, når jeg er irriteret	I eat more when I’m annoyed
6	Hunger	Jeg lægger ofte mærke til, at min mave rumler	I often notice my stomach rumbling
7	Food Fussiness	Jeg afviser nye typer mad i starten	I refuse new foods at first
8	Emotional Overeating	Jeg spiser mere, når jeg er bekymret	I eat more when I’m worried
9	Hunger	Hvis jeg springer et måltid over, bliver jeg irritabel	If I miss a meal I get irritable
10	Emotional Overeating	jeg spiser mere, når jeg er oprevet	I eat more when I’m upset
11	Satiety Responsiveness	Jeg levner ofte mad på min tallerken	I often leave food on my plate at the end of a meal
12 R	Food Fussiness	Jeg nyder at smage nye madvarer	I enjoy tasting new foods
13	Food Responsiveness	Jeg føler mig ofte sulten, når jeg er sammen med nogen der spiser	I often feel hungry when I am with someone who is eating
14 R	Slowness in Eating	Jeg bliver ofte hurtig færdig med at spise min mad	I often finish my meals quickly
15	Emotional Undereating	Jeg spiser mindre, når jeg er bekymret	I eat less when I’m worried
16	Emotional Overeating	Jeg spiser mere, når jeg er ængstelig	I eat more when I’m anxious
17	Food Responsiveness	Hvis jeg havde valget, ville jeg spise det meste af tiden	Given the choice, I would eat most of the time
18	Emotional Undereating	Jeg spiser mindre, når jeg er vred	I eat less when I’m angry
19 R	Food Fussiness	Jeg er interesseret i at smage ny mad, som jeg ikke har smagt før	I am interested in tasting new food I haven’t tasted before
20	Emotional Undereating	Jeg spiser mindre, når jeg er oprevet	I eat less when I’m upset
21	Emotional Overeating	Jeg spiser mere, når jeg er vred	I eat more when I’m angry
22	Food Responsiveness	Jeg tænker altid på mad	I am always thinking about food
23	Satiety Responsiveness	Jeg bliver ofte mæt før mit måltid er færdigt	I often get full before my meal is finished
24 R	Food Fussiness	Jeg kan godt lide mange forskellige slags mad	I enjoy a wide variety of foods
25	Slowness in Eating	Jeg er ofte sidst til at blive færdig med et måltid	I am often last at finishing a meal
26	Slowness in Eating	Jeg spiser mere og mere langsomt i løbet af et måltid	I eat more and more slowly during the course of a meal
27	Emotional Undereating	Jeg spiser mindre, når jeg er irriteret	I eat less when I’m annoyed
28	Hunger	Jeg føler mig ofte så sulten, at jeg må spise noget med det samme	I often feel so hungry that I have to eat something right away
29	Slowness in Eating	Jeg spiser langsomt	I eat slowly
30	Satiety Responsiveness	Jeg kan ikke spise et måltid, hvis jeg lige har spist en snack	I cannot eat a meal if I have had a snack just before
31	Satiety Responsiveness	Jeg bliver nemt mæt	I get full up easily
32	Hunger	Jeg føler mig ofte sulten	I often feel hungry
33	Food Responsiveness	Når jeg ser eller dufter mad, som jeg kan lide, får jeg lyst til at spise	When I see or smell food that I like, it makes me want to eat
34	Hunger	Jeg bliver svimmel, hvis mine måltider bliver forsinket	If my meals are delayed I get light-headed
35	Emotional Undereating	Jeg spiser mindre, når jeg er ængstelig	I eat less when I’m anxious

Original English wording and Danish translations of all items in the Adult Eating Behavior Questionnaire. The questionnaire measures appetitive traits in two domains: food approach (Hunger; Food Responsiveness; Emotional Overeating; Enjoyment of Food) and food avoidance (Satiety Responsiveness; Emotional Undereating; Food Fussiness; Slowness in Eating). Item numbers follow the original instrument; items marked “R” are reverse-scored. Content adapted from the original AEBQ developed by Hunot et al., 2016 [11].

## Appendix B

As a post hoc sensitivity analysis, we repeated the ICC analyses in participants with a retest interval ≤16 days (*n* = 133); results are shown in Table A3. The pattern of reliability was very similar to the main analysis, with all subscales in the moderate-to-excellent range and absolute differences in ICCs being small.

**Table A3:** Test–retest reliability (participants with ≤16 days between test and retest).

	Test 1	Test 2			
Subscale	Mean (SD)	Mean (SD)	SEM	MDC	ICC (95% CI)
Food Approach Subscales					
HU	2.78 (0.75)	2.76 (0.74)	0.319	0.885	0.815 (0.752; 0.866)
FR	2.85 (0.82)	2.82 (0.82)	0.318	0.882	0.849 (0.796; 0.891)
EOE	3.03 (1.03)	2.98 (1.07)	0.449	1.243	0.817 (0.754; 0.867)
EF	3.81 (0.85)	3.80 (0.82)	0.311	0.861	0.860 (0.810; 0.899)
Food Avoidance Subscales					
SR	2.81 (0.83)	2.82 (0.78)	0.344	0.955	0.816 (0.752; 0.866)
EUE	2.45 (0.89)	2.55 (0.89)	0.474	1.315	0.716 (0.626; 0.791)
FF	2.30 (0.91)	2.28 (0.86)	0.277	0.769	0.902 (0.865; 0.929)
SE	2.56 (0.92)	2.58 (0.89)	0.359	0.994	0.843 (0.787; 0.886)

Values are mean (SD) at Test 1 and Test 2. SEM = standard error of measurement; MDC = minimal detectable change; ICC = intraclass correlation coefficient; CI = confidence interval. HU = Hunger; FR = Food Responsiveness; EOE = Emotional Overeating; EF = Enjoyment of Food; SR = Satiety Responsiveness; EUE = Emotional Undereating; FF = Food Fussiness; SE = Slowness in Eating.
