# Supplementary material for: Validity and Reliability of the Danish Version of the Adult Eating Behavior Questionnaire—Results from the South Danish Obesity Initiative
Source: Nutrients. 2025 Dec 6;17(24):3824. doi: 10.3390/nu17243824 (PMC12736305; doi:10.3390/nu17243824)
Supplement: Supplementary file 1 [file nutrients-17-03824-s001.zip › nutrients-4006197-supplementary.pdf]

Supplementary Material for: "Validity and Reliability of the Danish version of the Adult Eating Behavior Questionnaire – Results from the South Danish Obesity Initiative"

Three-Step Test Interview: Overview and Focus Areas

The following section summarizes findings from the think-aloud phase of the Three-Step Test Interview (TSTI), which was used to evaluate the Danish translation of the Adult Eating Behavior Questionnaire (AEBQ). The aim was to assess how participants understood and related to the item content in real time, and to identify any potential issues in item interpretation or contextual relevance.

The analysis is structured around three core aspects of content validity, following COSMIN guide lines [1]:

- **Relevance**: whether participants judged the items to be pertinent to their experience and the intended construct/context.
- **Comprehensibility**: whether the instructions, items, and response options were clear and interpreted as intended.
- **Comprehensiveness**: whether all important aspects of the construct were covered (i.e., no key concepts missing)

Findings from this analysis are grouped thematically to illustrate the degree of alignment between item wording and participant interpretation, and to highlight areas where subtle semantic ambiguity or conceptual gaps were noted.

Comprehensibility and Relevance

Participants demonstrated adequate understanding of the translated questionnaire items, as evidenced by clear paraphrasing, elaboration, and reflective comments during the think-aloud portion of the Three-Step Test Interview. In particular, several items prompted spontaneous interpretations that aligned with the intended constructs, indicating good comprehensibility.

For example, when presented with the item "I refuse new foods at first", Participant ID 1 responded: "Strongly disagree… if I haven't tasted it before, then I have to try it", demonstrating a literal understanding of the item and endorsing the behavioral opposite. A similar interpretation was offered by Participant ID 2, who responded to "*I often decide that I don't like a food before tasting it*": "um no, I don't do that… I'm actually very open to trying everything… I'm usually quite good at tasting things before deciding whether I don't like them or not", again showing an accurate reading of the item and alignment with personal behavior. Participant ID 3 offered a

comparable elaboration, stating: "*I like tasting new food, so that would be disagree*", further supporting the comprehensibility of this item across individuals. Likewise, Participant ID 4 stated: *"I strongly disagree… I was raised to taste food before saying whether I like it or not*", grounding their response in both comprehension and social norms.

Similarly, the item "I often leave food on my plate at the end of a meal" elicited a response grounded in personal norms: *"Leaving food is wasteful according to our mother*", showing that the participant understood the item content and related it to personal values. Participant ID 4 provided a distinct behavioral rationale for agreeing with the same item, explaining: *"I always use small plates so the food feels like more, and that makes it easier to leave some"*, suggesting both item comprehension and relevance to personal eating strategies.

Items assessing internal states such as hunger and satiety also prompted meaningful elaboration. For "I often feel so hungry that I have to eat something right away", one participant responded: *"Disagree… I've practiced listening to myself, so it's rare that I get to that point",* demonstrating both accurate comprehension and reflective insight into personal regulation strategies. Participant ID 3 echoed this point with a temporal qualifier: "*Half a year ago I'd probably agree, but now I'm better at eating snacks, and that helps",* suggesting a clear understanding of the item's intent and a self-aware shift in behavior. Similarly, ID 4 commented: *"I can walk around hungry for a long time without needing to eat"*, illustrating the ability to interpret hunger-related items in context.

Emotional eating constructs also appeared to be well comprehended. Participant ID 2 stated in response to "I eat more when I'm worried": "*100%, I think most people do… you tend to find something to snack on or eat when you're worried… because it distracts you",* offering a direct and reflective elaboration consistent with the construct. Conversely, Participant ID 3 distinguished between emotional states in their response to "I eat more when I'm irritated": *"I don't feel that… maybe when I'm tired, but not irritated*", demonstrating clear differentiation between internal states and behavioral triggers, and supporting the cognitive clarity of the item set. Participant ID 4 similarly distinguished affective contexts, repeatedly stating that worry, irritation, and anger typically lead to eating less: "*I eat less when I'm angry… I can't concentrate on food when I'm angry"*. These responses validate that the emotional eating items were generally well understood, with variation in agreement reflecting behavioral diversity rather than misunderstanding.

Other participants also offered detailed insight into how they interpret terms like "enjoy". For example, ID 3 explained their response to "I enjoy eating" by stating: *"To enjoy… in my world that means sitting down and savoring the food, and I'm not good at that"*, showing an accurate yet personally situated understanding of item semantics. Participant ID 4 also affirmed enjoyment of food in broad terms: "I love all kinds of food", and expressed strong agreement with "I enjoy trying new foods", linking the items to active enthusiasm and exploratory behaviors.

Similarly, when evaluating "I often finish my meals quickly", a participant explained: *"I eat really slowly… to feel my fullness… it's very intentional. Learned"*, indicating both a correct interpretation of the item and intentional regulation of eating pace, aligning well with the underlying construct. In contrast, ID 4 noted that eating speed depended on context: "*It depends on what kind of food*

and when I last ate", suggesting appropriate judgment of situational relevance and limits of generalizability for that item.

In several cases, participants also reflected on the relevance of items. For instance, in response to "I am always thinking about food", one participant noted: "*I think a lot about planning… but thinking about eating: to a lesser degree"*, indicating an ability to differentiate between cognitive planning and food craving. Others introduced life-course perspectives, such as ID 3, who commented: "*I used to feel that way, but it's gotten better… since I got better at eating snacks"*, suggesting thoughtful assessment of personal behavior over time. Participant ID 4 similarly stated: *"I used to feel hungry more often, but I've gotten better at eating regularly",* reinforcing the idea that participants integrated past experience and behavioral change into their responses.

Participant ID 5 offered additional insight into situational and contextual relevance. When reflecting on "*I look forward to mealtimes"*, they responded: "*That one's harder… depends on whether you know what you're going to eat",* indicating cognitive engagement and conditional endorsement. In response to "I eat when others eat", they remarked: *"No, I don't feel hungry, but I still eat"*, suggesting social factors influence eating behavior despite the absence of hunger. For "I enjoy trying new foods", they agreed but qualified: "*Yes, if it looks okay"*, reflecting a nuanced endorsement tied to visual appraisal—supporting both item clarity and contextual flexibility.

Taken together, these examples indicate that the Danish translation of the AEBQ items was generally well understood and interpreted as intended. Responses elicited thoughtful engagement and appeared to reflect the underlying constructs of the original scale.

Discrepancies in Semantic Interpretation

While participants generally understood the item content as intended, some responses revealed subtle differences in how certain items were semantically interpreted. These minor discrepancies suggest that item wording can carry slightly varied connotations depending on individual framing and experiential context.

For example, in response to "I love food," several participants interpreted the item as referring not only to the act of eating but also to food preparation, presentation, or broader symbolic meanings. Participant ID 2 strongly agreed with the item and described food as both a way of experiencing the world and expressing oneself: "*Food is really a great way to express yourself, but also to experience the world – it's fantastic.*" In follow-up reflection, they emphasized that the types of food one prefers—and particularly the food one prepares—can reflect identity, noting that *"the way you cook food yourself can quickly explain who you are."*

Participant ID 3 also interpreted the item in relation to cooking and context. While selecting "agree," they noted: *"I like food, but I don't love cooking it,"* and reflected: *"If I were served it, I would probably say I loved food, but when I make it myself, then it's more like… I eat because I need to."* Here, the interpretation extended beyond eating to encompass effort, routine, and pleasure—suggesting that the meaning of "loving food" was shaped by how food was accessed and experienced.

ID 5, who responded “strongly agree,” similarly included cooking and aesthetics in their understanding. They stated that loving food involved liking to cook, enjoying eating, and making food both taste and look good: *“I like cooking and enjoying food… it should taste good,”* and added, “*It should also look good.”* For them, appreciation of food included sensory pleasure, creativity, and presentation.

Participant ID 4 also selected “strongly agree” but offered a broader and more inclusive perspective. They described loving food as being *“open to exploring food and what it can do,”* emphasizing variety, willingness to try new things, and contrasting it with those who experience eating as burdensome. They noted: “*Food can do everything… who doesn’t love food?”,* suggesting that for them, the item captured a general openness and positive orientation toward food, with less focus on specific acts like cooking or tasting.

In a related response, the same participant (ID 4) also endorsed the item “I think about food all the time,” but clarified: *“Agree… not because I crave it, but because our world revolves around food—how much to eat, how little to eat.”* This indicates an interpretation grounded in societal discourse or cultural saturation with food messaging, rather than personal preoccupation or craving. Their agreement with the item may therefore reflect perceived cultural relevance rather than a direct experiential alignment.

Together, these responses illustrate that even seemingly straightforward items can evoke layered or personally modulated meanings, potentially influencing how different respondents position themselves on the response scale.

Perceived Ambiguity Around “Anxious Eating”

One item that prompted hesitation was “I eat more when I’m anxious.” Participant ID 2 disagreed with the statement and noted that they interpreted “anxious” as synonymous with experiencing clinical anxiety or panic. They explained: “*If I have a panic attack, I don’t eat… my thoughts aren’t on food—it’s survival.”* This response suggests that the term “anxious” may have been understood as referring to more intense psychological states, rather than mild worry or restlessness.

Such variation in interpretation may be important to consider in translation, where words like ‘*ængstelig’* in Danish can carry strong clinical connotations for some respondents. However, this is not necessarily a language-specific issue. In English as well, “anxious” may be interpreted along a spectrum—from everyday unease to pathological anxiety—meaning the original item may already be vulnerable to variability in respondent interpretation. While the item is semantically correct, both its Danish and English versions may elicit different associations depending on individual experience and framing.

Comprehensiveness: Perceived Gaps in Affective Triggers

From a comprehensiveness perspective, participants identified affective or situational triggers they associate with increased eating that are not represented in the current item set. Participant ID 3, for example, disagreed with items such as “I eat more when I’m upset” or “when I’m angry,” explaining: “It’s more tiredness I eat from… that’s not mentioned anywhere.” They described post-

work exhaustion as a distinct trigger for sugar cravings, suggesting tiredness/fatigue may constitute a motivational domain not currently covered.

Similarly, Participant ID 5 remarked: “You don’t have anything about boredom,” adding, “If I don’t have anything to do, I end up looking in cupboards and fridges.” This points to boredom/low stimulation as a potential trigger independent of traditional negative emotional states.

These states—tiredness and boredom—are not part of the original Adult Eating Behavior Questionnaire. To preserve conceptual fidelity during translation and cross-cultural adaptation, we did not add items beyond the validated source instrument. Nonetheless, these insights may inform future iterations or culturally responsive extensions, particularly in populations where such triggers are salient.

Taken together, these reflections indicate a minor limitation in comprehensiveness of the emotional-eating construct as operationalized in the AEBQ. Future adaptations might consider incorporating additional affective/situational triggers to better capture respondents’ lived experiences.

Conclusion

Overall, the think-aloud interviews suggest that the Danish translation of the Adult Eating Behavior Questionnaire was comprehensible, meaningful, and conceptually aligned with participant experiences. Respondents demonstrated clear understanding of item content, frequently elaborating with examples that mapped onto the underlying constructs. While minor variation in interpretation and perceived content gaps were identified—particularly around certain affective states—these did not reflect fundamental misunderstandings but rather pointed to context-specific nuances and potential areas for future scale refinement. As such, the translated instrument appears to function as intended, eliciting reflective, construct-relevant responses across a diverse sample.

Reference

1. Gagnier JJ, Lai J, Mokkink LB, Terwee CB. COSMIN reporting guideline for studies on measurement properties of patient-reported outcome measures. Quality of life research. 2021;30(8):2197-218.
